# POLYGENIC RISK SCORES FOR ALZHEIMER’S DISEASE AND GENERAL COGNITIVE FUNCTION ARE ASSOCIATED WITH MEASURES OF COGNITION IN OLDER SOUTH ASIANS

**DOI:** 10.1093/geroni/igae098.0565

**Published:** 2024-12-31

**Authors:** Wei Zhao

**Affiliations:** University of Michigan, Ann Arbor, Michigan, United States

## Abstract

TBD

